# Application of vancomycin-impregnated calcium sulfate hemihydrate/nanohydroxyapatite/carboxymethyl chitosan injectable hydrogels combined with BMSC sheets for the treatment of infected bone defects in a rabbit model

**DOI:** 10.1186/s12891-022-05499-z

**Published:** 2022-06-09

**Authors:** Yanjun Wang, Zihou Zhao, Shiyu Liu, Wen Luo, Guoliang Wang, Zhenfeng Zhu, Qiong Ma, Yunyan Liu, Linhu Wang, Shuaikun Lu, Yong Zhang, Jixian Qian, Yunfei Zhang

**Affiliations:** 1 Department of Orthopaedics, Second affiliated hospital, Air Force Medical University, Xi’an, 710038 Shaanxi China; 2 Institute of Oral Tissue Engineering, Air Force Medical University, Xi’an, 710032 Shaanxi China; 3 Department of Ultrasound, Xijing Hospital, Air Force Medical University, Xi’an, 710032 Shaanxi China

**Keywords:** Nanohydroxyapatite, Calcium sulfate hemihydrate, Carboxymethyl chitosan, Bone marrow mesenchymal stem cells, Osteomyelitis

## Abstract

**Background:**

The choice of bone substitutes for the treatment of infected bone defects (IBDs) has attracted the attention of surgeons for years. However, single-stage bioabsorbable materials that are used as carriers for antibiotic release, as well as scaffolds for BMSC sheets, need further exploration. Our study was designed to investigate the effect of vancomycin-loaded calcium sulfate hemihydrate/nanohydroxyapatite/carboxymethyl chitosan (CSH/n-HA/CMCS) hydrogels combined with BMSC sheets as bone substitutes for the treatment of IBDs.

**Methods:**

BMSCs were harvested and cultured into cell sheets. After the successful establishment of an animal model with chronic osteomyelitis, 48 New Zealand white rabbits were randomly divided into 4 groups. Animals in Group A were treated with thorough debridement as a control. Group B was treated with BMSC sheets. CSH/n-HA/CMCS hydrogels were implanted in the treatment of Group C, and Group D was treated with CSH/n-HA/CMCS+BMSC sheets. Gross observation and micro-CT 3D reconstruction were performed to assess the osteogenic and infection elimination abilities of the treatment materials. Histological staining (haematoxylin and eosin and Van Gieson) was used to observe inflammatory cell infiltration and the formation of collagen fibres at 4, 8, and 12 weeks after implantation.

**Results:**

The bone defects of the control group were not repaired at 12 weeks, as chronic osteomyelitis was still observed. HE staining showed a large amount of inflammatory cell infiltration around the tissue, and VG staining showed no new collagen fibres formation. In the BMSC sheet group, although new bone formation was observed by gross observation and micro-CT scanning, infection was not effectively controlled due to unfilled cavities. Some neutrophils and only a small amount of collagen fibres could be observed. Both the hydrogel and hydrogel/BMSCs groups achieved satisfactory repair effects and infection control. Micro-CT 3D reconstruction at 4 weeks showed that the hydrogel/BMSC sheet group had higher reconstruction efficiency and better bone modelling with normal morphology. HE staining showed little aggregation of inflammatory cells, and VG staining showed a large number of new collagen fibres.

**Conclusions:**

Our preliminary results suggested that compared to a single material, the novel antibiotic-impregnated hydrogels acted as superior scaffolds for BMSC sheets and excellent antibiotic vectors against infection, which provided a basis for applying tissue engineering technology to the treatment of chronic osteomyelitis.

**Supplementary Information:**

The online version contains supplementary material available at 10.1186/s12891-022-05499-z.

## Background

Infected bone defects (IBDs) include both infection and defects, which are mutually causative. The sequestrum formation after infection leads to bone defects. Moreover, large defects are not conducive to local infection control, and the formation of haematomas in defects can create opportunities for bacterial breeding [1]. The high incidence of IBDs is due to factors such as osteomyelitis, traumatic events, and iatrogenic factors [2, 3]. Effective treatment should not only eliminate infection but also repair defects to alleviate the pain of patients and restore the normal function of limbs. Traditionally, the treatment strategy for IBDs can be summarized as follows: control and elimination of infected factors, maintenance of fracture stability, reconstruction of bone defects, and proper functional exercise after surgery [4, 5]. Thorough debridement and defect reconstruction can be carried out either in one stage or two according to the site, scope, and severity of infection [6].

Recently, studies on single-stage bioabsorbable bone substitute fillings used as carriers for antibiotic release while repairing defects after debridement, which can overcome both the limited supply of autologous bone and the risk of disease transmission of allograft bone, have attracted the interest of surgeons [7–9]. Artificial synthetic bone substitutes, such as calcium sulfate hemihydrate (CSH) and nanohydroxyapatite (n-HA), are reasonable alternatives, as they are biodegradable and osteoconductive with excellent biocompatibilities. In addition, they can also be used as vectors for antibiotic release. Although the biomechanical strength of CSH is higher compared to that of CS alone, its absorption rate is still faster than the rate of new bone regeneration. n-HA has a low absorption rate but a higher solubility, stronger surface energy, and greater absorbability than HA. A combination of CSH and n-HA, which exhibits synergistic osteogenic properties, overcomes the deficiencies of the two materials alone [10, 11]. The introduction of the organic material carboxymethyl chitosan (CMCS) makes up for the weaknesses caused by inorganic materials, such as Ca^2+^ or PO_4_^3^-, easily increasing local pH [12, 13]. This material not only enables composites to regulate immunity but also increases their stability and sustained antibiotic release efficiency as well as simulates the natural composition of the bone.

Cell sheet technology (CST) was first proposed by Japanese professor OKANO in 1993 [14]. The greatest advantages of this technique are that it does not require enzymes to acquire cultured cells and preserves the extracellular matrix (ECM), cell-to-cell adhesion proteins, ion channels and growth factor receptors [15–18]. BMSCs have the potential for multidirectional differentiation and rapid proliferation and can be directed to differentiate into chondrocytes and osteocytes. Constructing BMSCs into a cell sheet in vitro enables biocompatibility and a high inoculation rate, and this cell sheet also inherits the osteogenic ability of BMSCs. Maniatopoulos et al. first reported the use of BMSCs to induce osteogenesis in vitro in 1988 [19]. Recent studies have demonstrated the feasibility of combining BMSC sheets with HA particles to induce new bone formation in vivo [20, 21].

A local antibiotic delivery system is preferred over intravenous antibiotics because of its lower cytotoxicity (including nephrotoxicity and ototoxicity) and longer maintenance of antibiotic release. Various kinds of antibiotics are available when loaded with bone substitutes, and among them, vancomycin tends to be a better choice despite its narrow antimicrobial spectrum [11, 22]. First, water-soluble vancomycin is dose-dependent and is effective against the most common *Staphylococcus aureus* infections of osteomyelitis. Furthermore, it has the lowest cytotoxic effect, and normal cell activity is only affected when the local concentrations are too high. In addition, vancomycin is one of the few antibiotics that can inhibit bacteria released from biofilms [23, 24]. However, no studies have evaluated the validity of vancomycin-loaded CSH/n-HA/CMCS hydrogels combined with BMSC sheets for the treatment of IBDs in a rabbit model.

In our study, a new type of vancomycin-loaded CSH/n-HA/CMCS hydrogels is injectable as well as biodegradable, which is attributed to the mechanism of cross-linking of the Schiff-base reaction between the amino and aldehyde groups of the carboxymethyl chitosan (CMCS) and oxidized alginate. A combination of organic and inorganic materials not only endows the hydrogel with proper mechanical properties, a suitable degradation rate, osteogenesis, and properties of antibiotic loading but also preserves the extracellular matrix and growth factors of BMSCs with multidirectional differentiation potential through the introduction of BMSC sheets. VA/CSH/n-HA/CMCS hydrogels combined with BMSC sheets are applied in the treatment of focal infected defects of rabbit tibia. Radiographic evaluation (micro-CT 3D reconstruction) and histological techniques are applied to detect and evaluate infection control and bone defect restoration to provide theoretical support for the selection of bone substitutes and antibiotics in the clinical treatment of focal infected bone defects.

## Methods

### Preparation of BMSC sheets

BMSCs were isolated and obtained under sterile conditions from both sides of the femurs of 10-week-old New Zealand white rabbits and used to create single-cell suspensions. Then, the adherent culture method was adopted. Cells were cultured in the air with 5% CO_2_ at 37 °C. The cells at passage 1 were digested by trypsin when 90% of cells reached confluence, and the 3rd generation of high purity was obtained by the same method for subsequent experiments. To harvest BMSC sheets, the cells at passage 3 were seeded onto culture dishes at a density of 9 × 10^4^ cells/dish. When 75% of the cells reached confluence, DMEM (containing 10% FBS, 100 mg/L L-ascorbic acid, 100 unit/ml penicillin G, 100 unit/ml streptomycin, and 2 mmol/l L-glutamine) was used and cultured continuously for 15 days until the sheet was sufficiently thick. The sheet was rinsed twice with powdered PBS and then scraped with a cell scraper.

Experiments were then performed to identify and detect the characteristics of the BMSCs. The cells at passage 3 must meet the following criteria to be defined as BMSCs according to the rules of the Mesenchymal and Tissue Stem Cell Committee of the International Society for Cellular Therapy (ISCT): plastic adherence in standard media, expression of specific surface markers (CD73+, CD90+, CD105+, CD45-, CD34-, CD14-, CD19-, and HLA-DR-), and in vitro differentiation potential into osteocytes, adipocytes, and chondrocytes. Flow cytometry was used to detect the specific markers of the BMSCs. The BMSCs at passage 3 were inoculated on culture dishes at a density of 1 × 10^5^ cells/cm^2^. Osteogenic induction medium (10% FBS, 100 mg/L L-ascorbic acid, 100 unit/ml penicillin G, 100 unit/ml streptomycin, 2 mmol/l L-glutamine, 10 mmol/L dexamethasone and 10 mmol/L sodium glycerophosphate) was used for culture at 37 °C in a 5% CO_2_ incubator. The nutrient solution was changed every 3 days for up to 21 days. Alkaline phosphatase (ALP) staining was performed at 7, 14, and 21 days after cultivation. For adipogenesis, Oil Red O staining was applied after cultivation with adipogenesis medium (1 μmol/L dexamethasone, 10 mg/mL insulin, and 500 mmol/L 1-methyl-3-isobutyl-xanthine) for 21 days. The medium was replaced every 3 days. The staining results were observed under a microscope and subsequently photographed.

### Hydrogel fabrication and characteristic detection

Calcium sulfate hemihydrate, nanohydroxyapatite, and carboxymethyl chitosan were purchased from Macklin Biochemical Co., Ltd. (Shanghai, China), and the hydrogels were manufactured based on Schiff conjugate crosslinking theory. The ambient temperature was adjusted to 25 °C to ensure the successful preparation of hydrogels. Briefly, 4 g of carboxymethyl chitosan was slowly added to 100 ml of purified water, and magnetic stirring was applied to prepare a 4% (w/v) carboxymethyl chitosan solution, followed by adding 30 mg of vancomycin. Then, 3.75 g of sodium alginate oxide was added to 25 ml of purified water and placed in a constant temperature shaker until the flocculent precipitate completely disappeared to prepare a 15% (w/v) solution. The ultrasonic dispersion method was used after adding CSH and 6% n-HA complex. Thus, a uniform and stable solution of sodium alginate oxide containing CSH/n-HA was obtained. Carboxymethyl chitosan solution was mixed with sodium alginate oxide at a ratio of 4:1 (v/v). Finally, the mixture was poured into a 5 mm × 10 mm cylindrical mould, placed in an incubator at 37 °C for 40 mins to obtain hydrogels, and sterilized with ethylene oxide.

Scanning electronic microscopy (SEM, Hitachi, Japan) was used to observe the microstructures and porosities of the hydrogels. Bacterial inhibition zone test: Briefly, 100 μl of *Staphylococcus aureus* (BeNa Culture Collection, Bei Jing, China) suspension at a concentration of 10^7^–10^8^ CFU/ml was evenly coated in Luria-Bertani (LB) solid medium. Then, the manufactured hydrogels were pasted on the medium and cultured at a temperature of 37 °C with the culture dish inverted. Eighteen hours later, bacterial growth was judged by measuring the diameter of the inhibition zone with a Vernier calliper. Then, the samples were transferred to a new medium containing *Staphylococcus aureus* for sequential culture. The medium was changed after measuring the zone once a day until it disappeared. Images were photographed digitally.

### Establishment of the animal model

A total of 48 New Zealand white rabbits provided by the experimental animal centre of Air Force Medical University were included in the study, half male and half female. Each weighed between 4.5–5.5 kg. All animal processes were approved by the institutional animal care and use of Tangdu Hospital, Air Force Medical University (IACUC-20200355), and were conducted in an open system laboratory with a temperature of 25 °C and relative humidity of approximately 40%. The establishment of an animal model of rabbit chronic osteomyelitis followed the Norder method [25]. Sodium pentobarbital (1 ml/kg, body weight) was injected into the rabbits through the ear vein. Then, the rabbits were placed in a supine position on the operating table and fixed well. We selected the right tibia as the predetermined position, and after shaving, iodophor was used for disinfection, and sterile tablecloths were set. We made a 3 cm incision along the medial side of the tibia, and a 12 mm × 6 mm bone window was created with an electric drill and oscillating saw directly into the medullary cavity. A total of 0.1 ml of 5% sodium glycolate and 3 × 10^7^ CFU/ml of *Staphylococcus aureus* in 0.2 ml of nutrient solution were injected into the cavity. To prevent bacterial leakage, bone wax was used to block the hole followed by irrigation with sterile saline. Rabbits were individually caged and fed standardized fodder.

### Surgical procedure

**Fig. 1:**
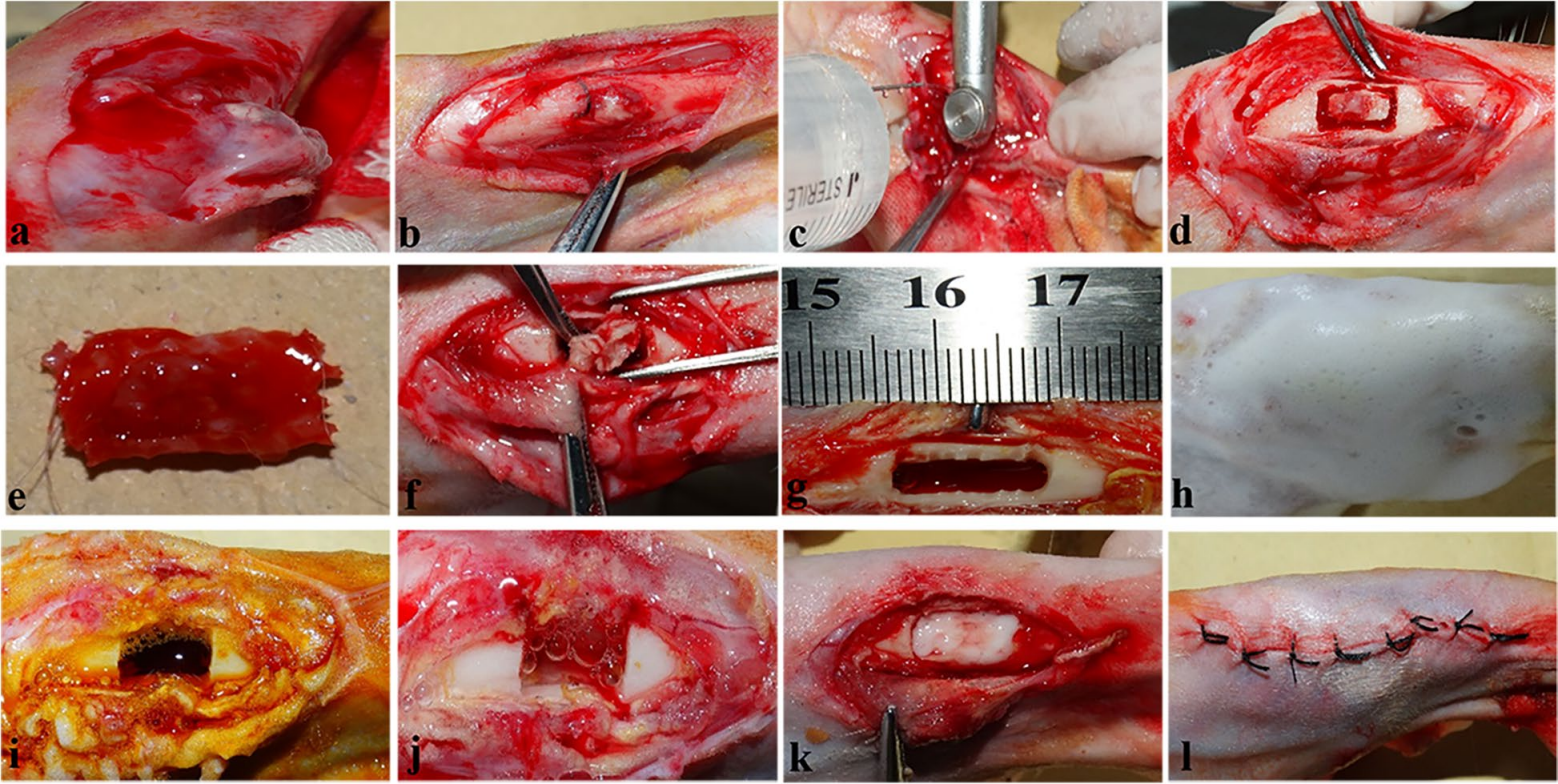
Surgical procedure of thorough debridement and filling of hydrogels after successful establishment of the animal model. The sinus tract and infected soft tissues were eliminated layer by layer and necrotic bone was removed by laminectomy rongeur (**a**-**e**). The bone marrow cavity was thoroughly cleaned (**f**), after which the size of the defect was measured (**g**). After being irrigated with hydrogen peroxide, iodophor, and sterile saline for 3 times (**h**-**j**), the corresponding materials were added and the incision was sutured (**k**, **l**)

The animal model of chronic osteomyelitis was successfully established 28 days later. Then, the 48 rabbits were allocated into 4 groups according to the randomization principle. General anaesthesia was applied to all operations, and we performed thorough debridement of all experimental animals. Rabbits were placed in a supine position, and the target site (right hind leg) was shaved and sterilized with iodophor. The skin was incised along the original incision until the infected defect was exposed. The sinus tract was eliminated, and full debridement of the soft tissue was performed layer by layer. Necrotic bone was removed by laminectomy rongeur, as well as pus from the intramedullary cavity. The defect was irrigated with sterile saline 2–3 times and soaked in dilute iodophor saline for 5 mins, after which the sterile tablecloths and gloves were replaced. In Group A (*n* = 12), we ignored the defect as a blank control. In Group B (*n* = 12), BMSC sheets were used as filler. In Group C (*n* = 12), the defect was treated with VA/CSH/n-HA/CMCS hydrogels, and animals in Group D (*n* = 12) were implanted with VA/CSH/n-HA/CMCS/BMSC sheet hydrogels. The wound was sutured, and analgesic was instituted after the operation. Gross observation, radiographic evaluation, and histological staining were performed at 4, 8, and 12 weeks after the operation. The operation process was as follows (Fig. 1).

### Gross observation

Three experimental animals in each group were euthanized, and gross tibial specimens were collected to assess soft tissue condition, callus formation, and whether chronic osteomyelitis still persisted at 4,8, and 12 weeks postoperatively.

### Imaging measurement

Postoperatively, 3 rabbits in each group were selected for micro-CT evaluation to acquire 3D images at 4, 8, and 12 weeks. After routine anaesthesia with sodium pentobarbital (1 ml/kg, body weight), animals were immobilized on the operating table. Tibia samples around the defect were carefully collected, and the surrounding soft tissue was removed and soaked in 4% paraformaldehyde. A micro-CT imaging system (μ CT 40, Siemens, Germany) was used to evaluate infection control, hydrogel absorption, and bone formation. The long axis of the tibial sample was perpendicular to the X-ray beam during scanning by fixing it on a proper holder. The target area (voxel size: 200 × 80) for evaluation was determined to contain no cortical bone and adhere to bone substitutes. A total of 1536 images were obtained from each sample and were measured by imaging experts. The reconstructed 3D images obtained were analysed using Inveon Research Workplace 2.2 software. Quantitative indicators of the bone volume to total volume ratio (BV/TV), trabecular thickness (Tb. Th), and trabecular number (Tb. N) were collected for the assessment of bone formation and material absorption.

### Histological evaluation

Haematoxylin and eosin (HE) staining was used qualitatively and quantitatively to detect whether inflammatory cell infiltration around tissues existed, and Van Gieson staining (VG) was used to observe the formation of collagen fibres. The tibia samples of each group were first fixed by soaking in 10% neutral formaldehyde for 2 days. Subsequently, the samples were decalcified with 10% ethylenediaminetetraacetic acid (EDTA, pH 7.0) and dehydrated with gradient concentrations of alcohol (70, 80, 90, 100%). The sections were embedded in methyl methacrylate and sectioned at 10 μm with a microtome (Leica RM2235 microtome, Leica Microsystems, Germany) for HE staining. Another group of harvested samples that were not decalcified after fixation were routinely dehydrated and embedded for 21 days. Sections were cut with a LA2500 (Leica Microsystems, Germany) at 10 μm for VG staining.

### Statistical analysis

SPSS statistics software (Version 23. 0; International Business Machines Corporation, Armonk, NY, USA) was used for data analysis. All quantitative data are presented as the mean ± standard deviation. One-way analysis of variance (ANOVA) was conducted to data conforming to normal distribution and homogeneity of variance. The Kruskal-Wallis H test was performed if the data failed the test of normality or homogeneity of variance in this study. *P* values < 0.05 were considered to be significant. All results were analysed by statisticians unrelated to the experiment.

## Results

### Morphological characteristics and identification of BMSCs

**Fig. 2:**
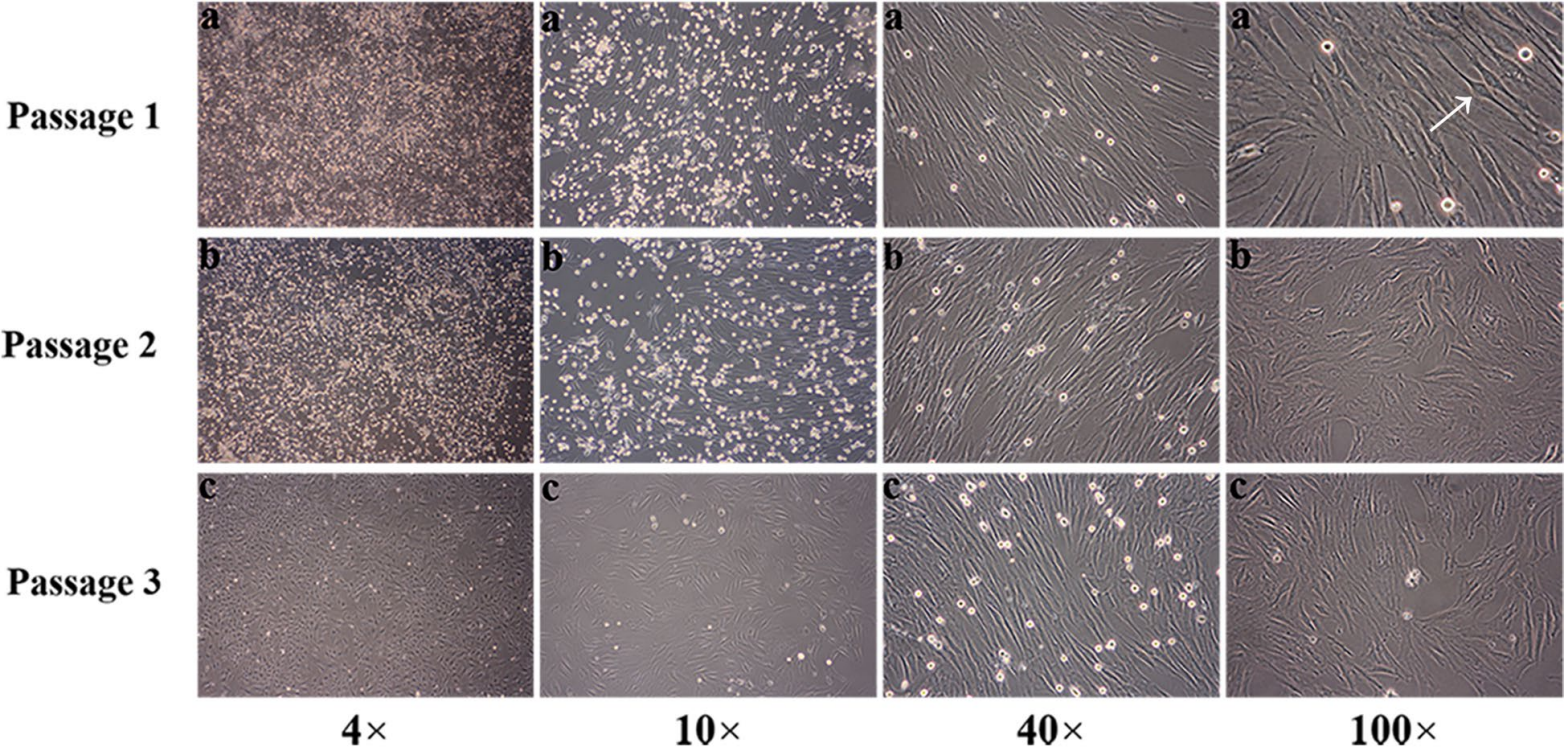
Morphology of cultured BMSCs under different magnifications of the microscope. Cells were cultured for 7 days and were short, spindle-shaped, polygonal, and storiform with vigorous growth (**a**). After primary culture (the 2nd passage), adherent cells began to appear in 24 hours and gradually increased in 48 hours with higher density and meaningful colonies (**b**). After passage culture (the 3rd passage) for 7 days, the cells presented as long spindles that were helically shaped and proliferated rapidly with a uniform structure and high purity (**c**)

The obtained BMSCs were maintained for 7 days in primary culture until 80 to 90% of the cells reached confluence and then subcultured. Adherent cells began to appear at 24 hours and gradually increased at 48 hours. By using different magnifications of optical microscopy, the following was demonstrated: the cells at passage 1 were short, spindle-shaped, polygonal, and storiform with vigorous growth (Fig. 2a), while the cells at passage 2 had higher density, and meaningful colonies appeared as well as stable proliferation (Fig. 2b), and the cells at passage 3 were long, helically shaped spindle cells and proliferated rapidly with a uniform structure and high purity (Fig. 2c).

**Fig. 3:**
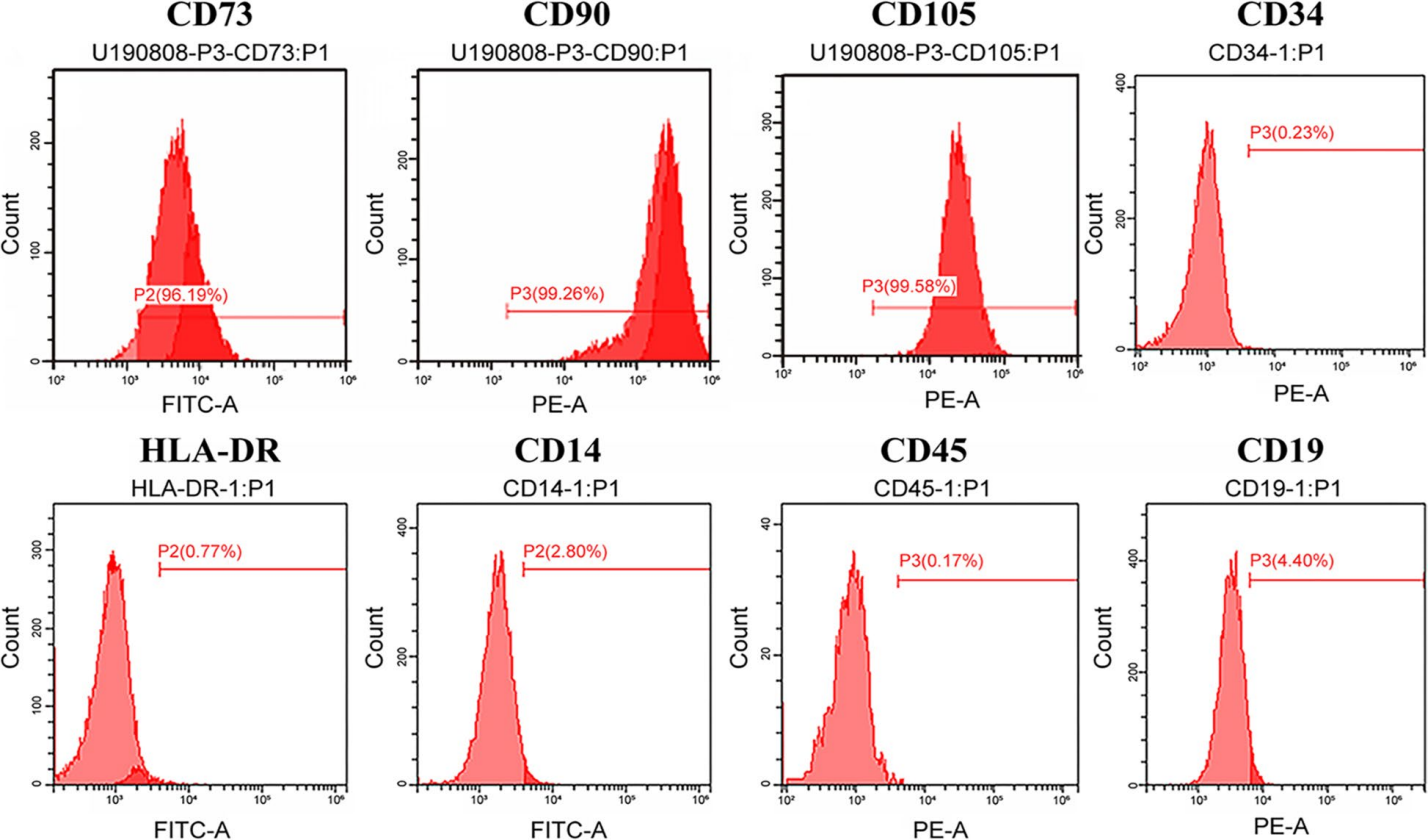
Results of the flow cytometry detection of BMSCs (the 3rd passage). **a**. CD73 (+); **b**. CD90 (+); **c**. CD105 (+); **d**. CD34 (−); **e** HLA-DR (−); **f** CD14 (−); **g** CD45 (−); **h** CD19 (−)

Surface antigen expression (including CD73, CD90, CD105, CD34, HLA-DR, CD14, CD45 and CD19) was detected by flow cytometry. We found that the 3rd passage of cultured cells was positive for CD73 (96.19%), CD90 (99.26%), and CD105 (99.58%), whose presentation rates were all over 95%, while they were negative for CD34 (0.23%), HLA-DR (0.77%), CD14 (2.8%), CD45 (0.17%) and CD19 (4.4%), which confirmed that the 3rd generation of cultured cells comprised BMSCs rather than HSCs (Fig. 3).

**Fig. 4:**
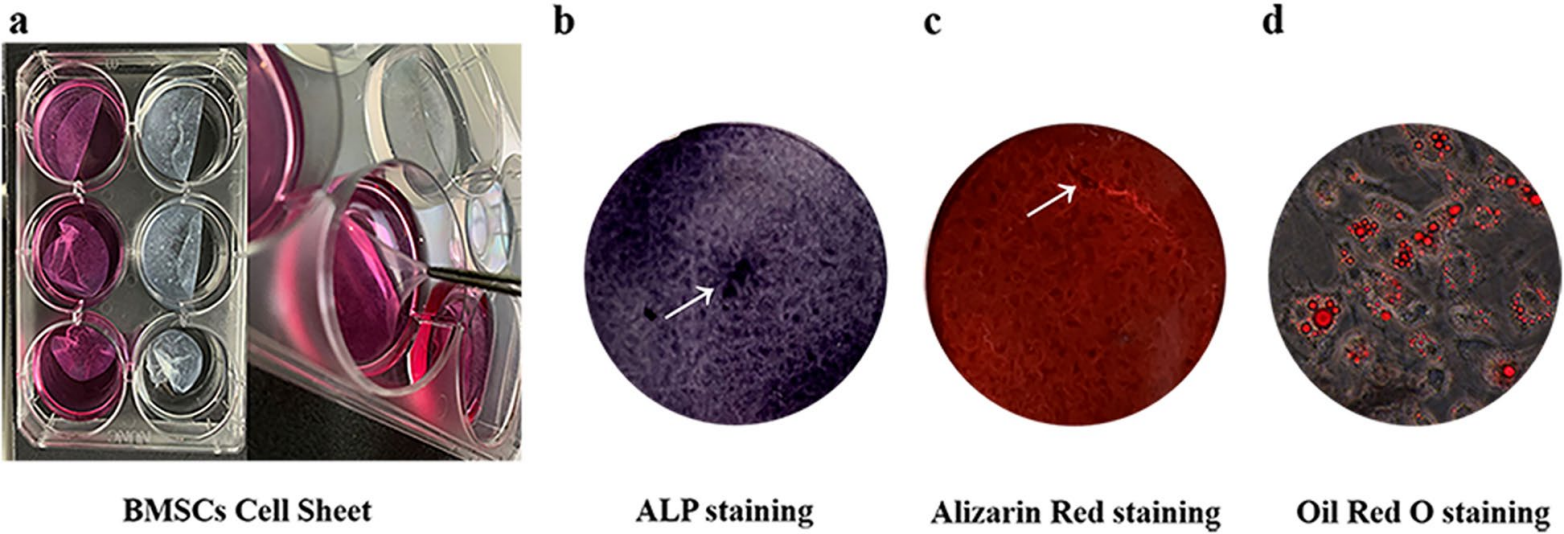
Cell sheets were obtained 14 days after the in vitro culture of BMSCs (passage 3). The cell sheets were translucent with a certain thickness and had a certain mechanical strength and elasticity (**a**). Alkaline phosphatase (ALP) staining of BMSCs after osteogenic induction showed positive staining, with the cytoplasm presenting blue and black staining (**b**), Alizarin Red staining showed typical mineralized nodules with orange red staining (**c**), and lipid droplets stained with Oil Red O showed orange red staining under a microscope (**d**)

Fourteen days after the culture of the BMSCs (Passage 3), a white translucent sheet-like substance with a certain thickness could be found at the bottom of the petri dish, which had a certain mechanical strength and elasticity and could be curled or folded. The cell sheet could be gently peeled off with sterilized ophthalmic forceps (Fig. 4a). After 14 days of the osteogenic induction of the BMSCs (passage 3), ALP staining was positive, with the cytoplasm presenting blue and black colours (Fig. 4b). After 21 days of induction, Alizarin Red staining showed typical mineralized nodules (Fig. 4c), and Oil Red O staining of lipogenetically differentiated BMSCs showed that lipid droplets in the cytoplasm were stained with orange red (Fig. 4d).

### Characteristics of VA/CSH/n-HA/CMCS hydrogels

**Fig. 5:**
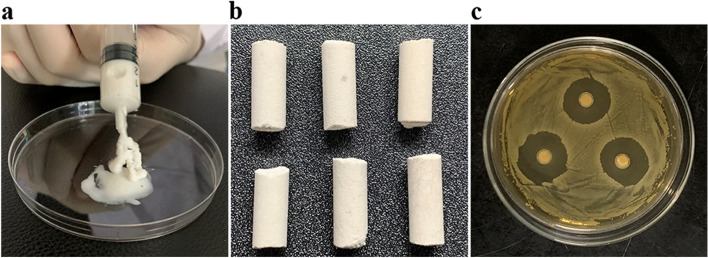
Characteristics of the synthetic VA/CSH/n-HA/CMCS hydrogel. It was injectable with good fluidity and was lyophilized into a cylindrical shape for observation with SEM (**a**, **b**). An inhibition zone for *Staphylococcus aureus* with an average diameter of 15 mm could be observed (**c**)

Figure 5a presents images of the white hydrogel scaffold after synthesis, showing that it is injectable with good fluidity. The hydrogels were lyophilized into cylindrical shapes for sectioning by a specific mould only for observation of the microstructure with scanning electron microscopy (SEM) (Fig. 5b). When the manufactured hydrogels were pasted on the medium and cultured at a temperature of 37 °C with the culture dishes inverted for 18 hours, we observed an obvious inhibition zone for *Staphylococcus aureus* with an average diameter of 15 mm, indicating that the gel had a good inhibition effect on SA (Fig. 5c).

**Fig. 6:**
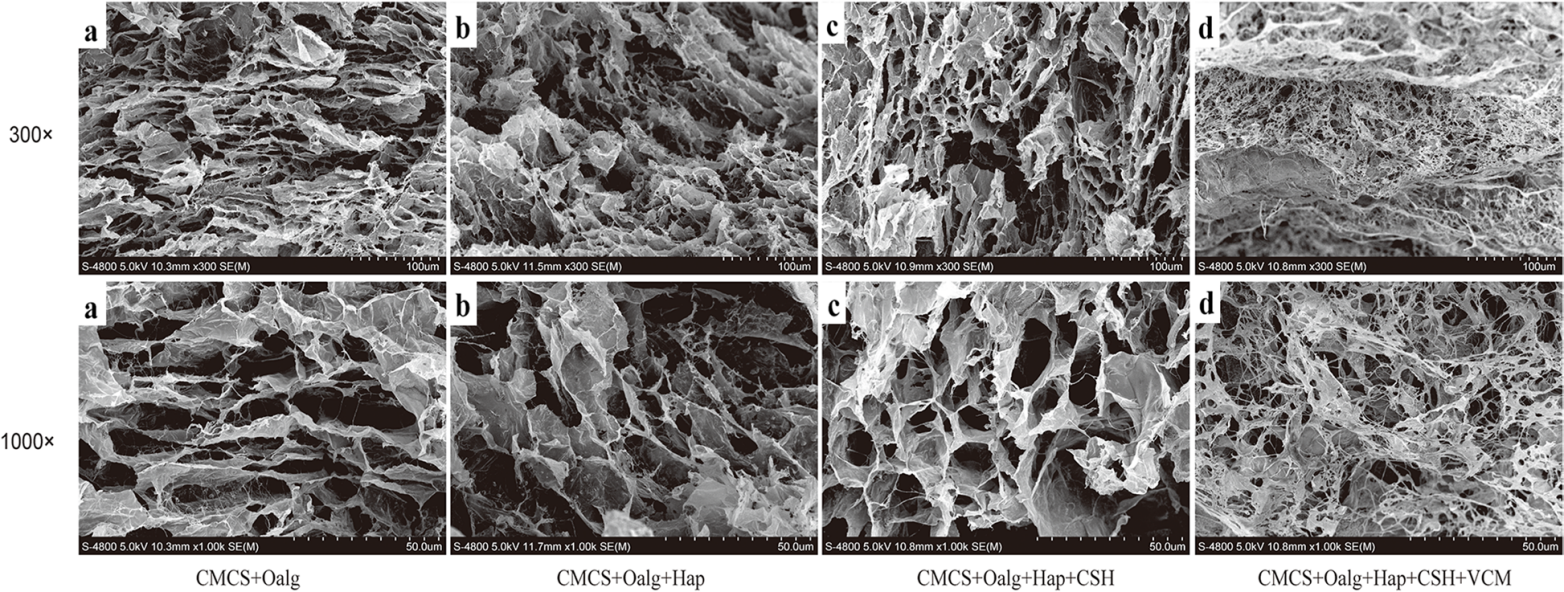
Scanning electron microscope (SEM) observation (300×, 1000×). The pore size was 80–200 μm, with an average of 140 μm, and mineral crystallization deposition could be observed in the pores of the VA/CSH/n-HA/CMCS hydrogel

The SEM micromorphologies of the hydrogels with different materials after freeze-drying microtomy are shown in Fig. 6. Generally, the scaffold materials still maintained a continuous three-dimensional network structure at low magnification (300×), while the pores were interpenetrating with good connectivity, and the pore size was 80–200 μm, with an average of 140 μm (Fig. 6a, b). With the introduction of the intensifying phase and vancomycin, the order and size of the pores decreased, gradually inducing disorder from the original regular arrangement, indicating that the introduction of CSH/n-HA and vancomycin also promoted the cross-linking of the hydrogels, which resulted in more compact network structures. In addition, it could be observed that the hydrogels had more porous structures due to the wide distribution of VA, and this right-sized aperture also provided proper space for new bone to grow in (Fig. 6c, d). Moreover, due to the addition of n-HA and CSH, mineral crystallization deposition could be observed in the pores of the composite scaffold, which appeared very rough (Fig. 6c). Higher porosity contributed to BMSC ingrowth and vascularization.

### Successful establishment of animal models

**Fig. 7:**
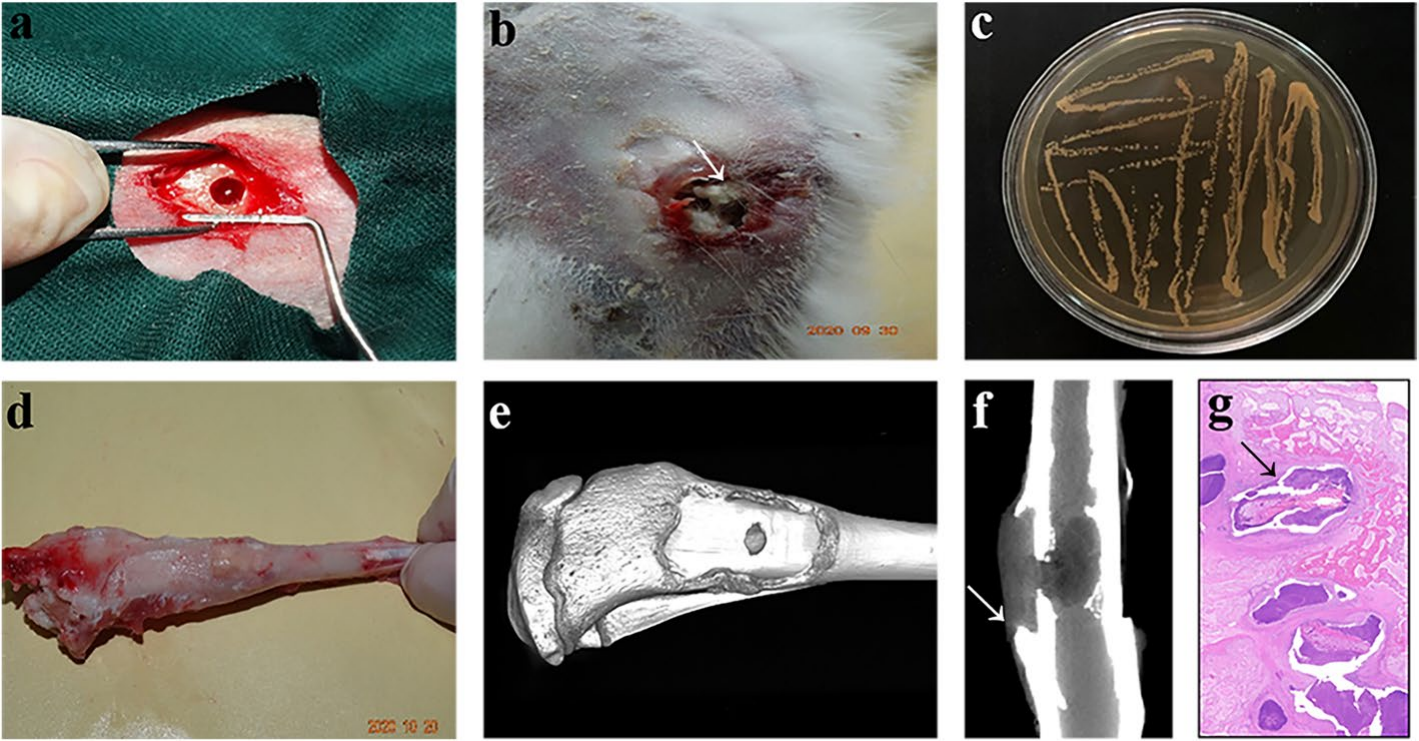
Establishment of rabbit models with chronic osteomyelitis. Gross observation, bacterial culture, micro-CT, and HE staining were carried out indicating typical characteristics of chronic osteomyelitis

Four weeks after the preparation of the chronic osteomyelitis model of the rabbits, all wounds produced pus (Fig. 7a, b), with local soft tissue swelling and lameness of the injured limb, accompanied by various extents of anorexia and weight loss. Bacterial cultures were positive for *Staphylococcus aureus* (Fig. 7c). Three patients died because of bacteraemia 2 weeks postoperatively, while the rest of the models in radiology were characterized by typical osteomyelitis 4 weeks later. Gross observation showed sinus tract formation (Fig. 7d). Radiographic examination revealed moderate to extensive cortical reactions, cortical bone destruction, and new bone formation (Fig. 7e, f). Osteomyelitic sequestration could be observed in the proximal tibial medullary cavity, and pathological examination revealed chronic active inflammation with bone dissolution and new fibrous bone formation (Fig. 7 g).

#### Gross observation

**Fig. 8:**
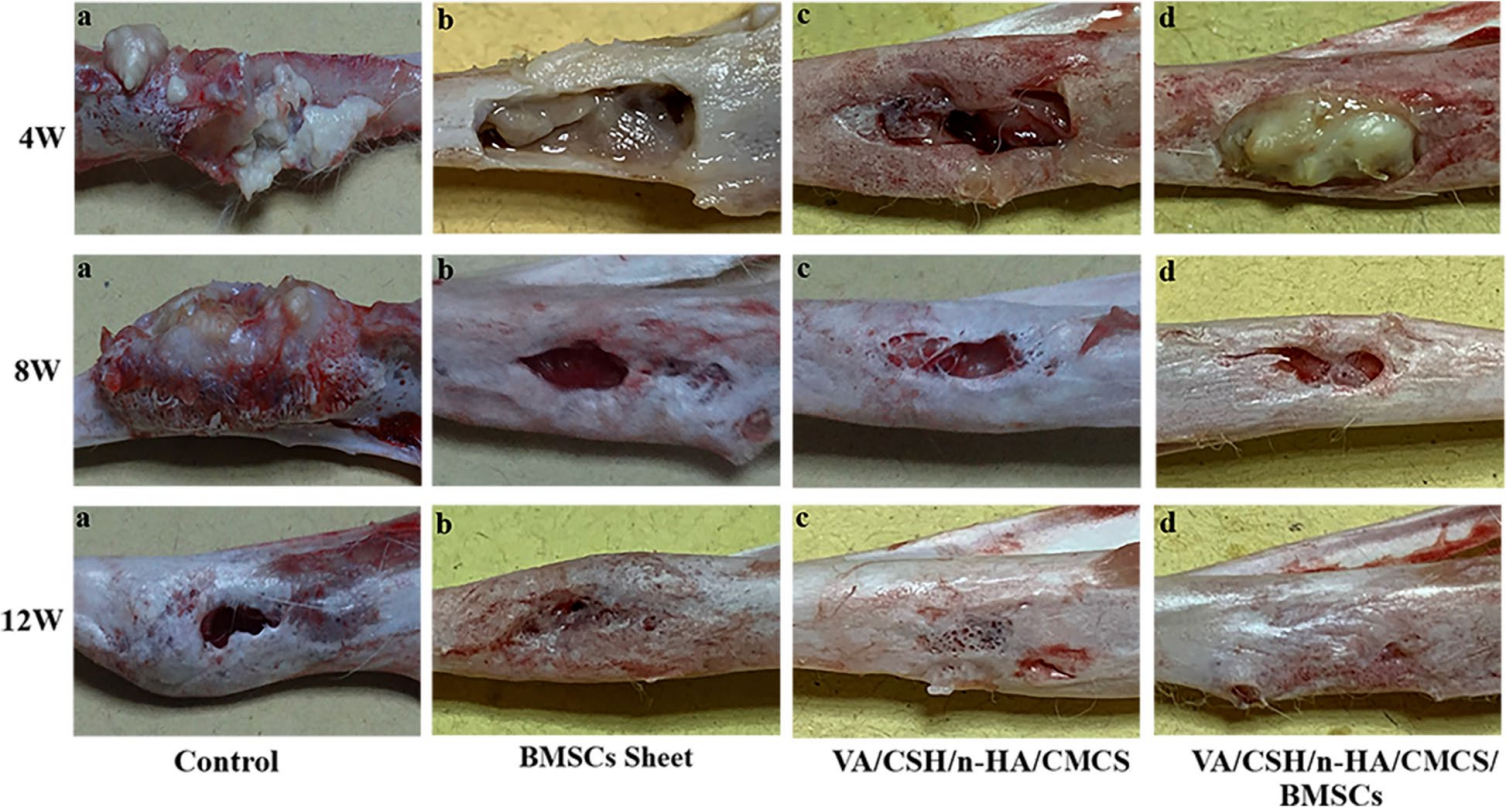
Morphological evaluation of each group by gross observation at 4 weeks, 8 weeks, and 12 weeks postoperatively. In the control group, typical osteomyelitis and severe tibia damage could be seen (**a**), and in the BMSC sheets group, the defects were partially healed with uncontrolled infection (**b**). It could be observed that defects were gradually cured with the absorption of bone substitutes, fibrous tissue was formed, and infection was effectively controlled in Groups C and D (**c**, **d**)

The right tibia samples of the four groups were harvested for gross observation at 4 weeks, 8 weeks, and 12 weeks after thorough debridement and planned treatment. The control group showed typical osteomyelitis and severe tibia damage at 4 weeks and 8 weeks. The bone defects remained unhealed with deformities of the tibias at 12 weeks (Fig. 8a). In the BMSC sheets group, the defects appeared to be as obvious as they originally were, but over time, they were partially healed, although the infection was uncontrolled (Fig. 8b). In Group C treated with VA/CSH/n-HA/CMCS, infection was eliminated, and the defects were restored much more completely than those in Group B (Fig. 8c). In Group D treated with VA/CSH/n-HA/CMCS/BMSC sheets, a clear boundary existed between the hydrogels and the bone tissues (Fig. 8d). As the bone substitutes were absorbed, abundant fibrous tissue was formed, and the defect cavities gradually decreased with novel bone formation; moreover, infection was effectively controlled. The defect was almost completely reconstructed 12 weeks postoperatively.

#### 3D reconstruction of micro-CT evaluation

**Fig. 9:**
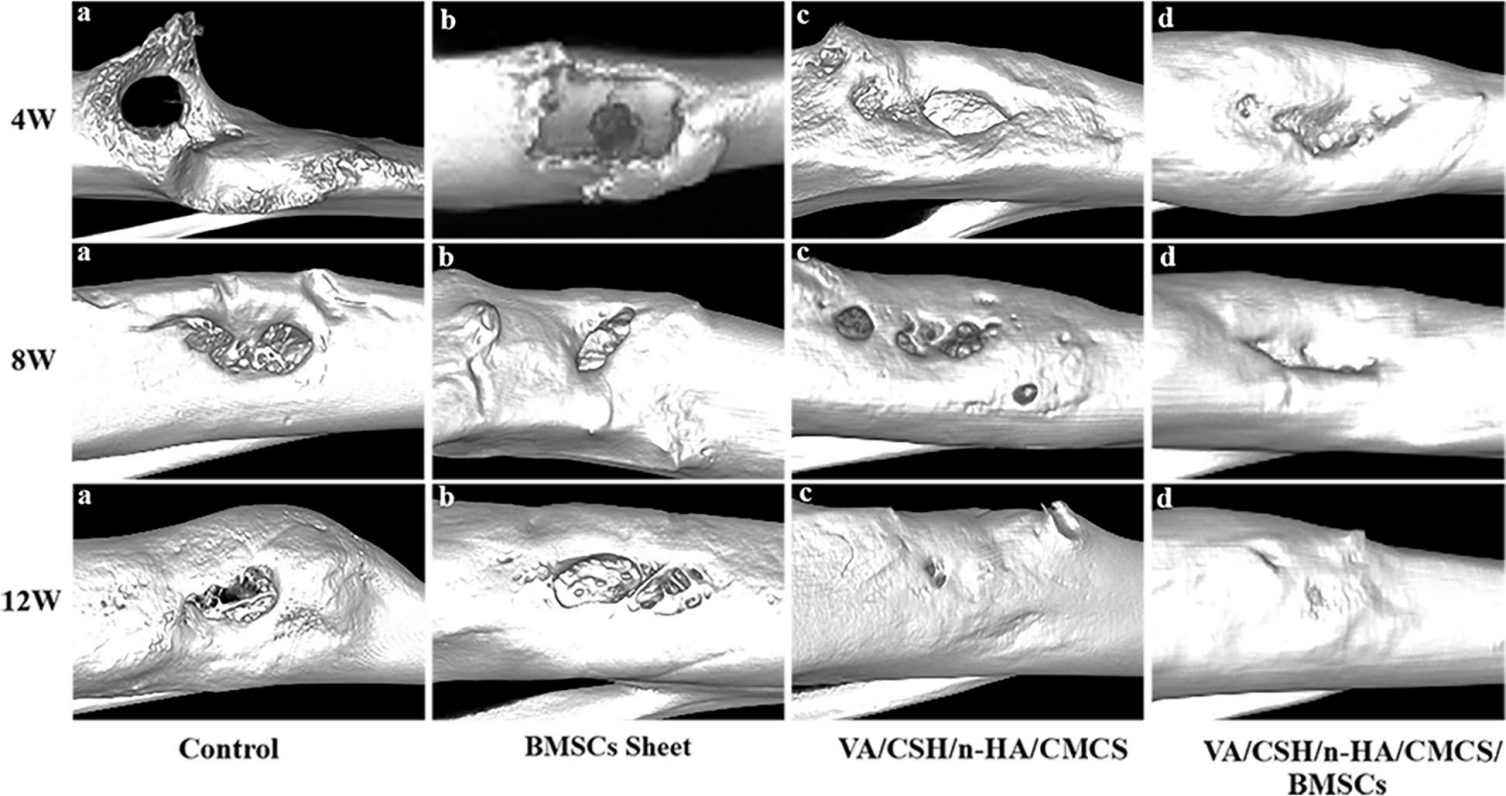
3D reconstruction images of the right tibias in each group at 4 weeks, 8 weeks, and 12 weeks after treatment. Reconstruction of defects and infection control were assessed at different time points

**Fig. 10:**
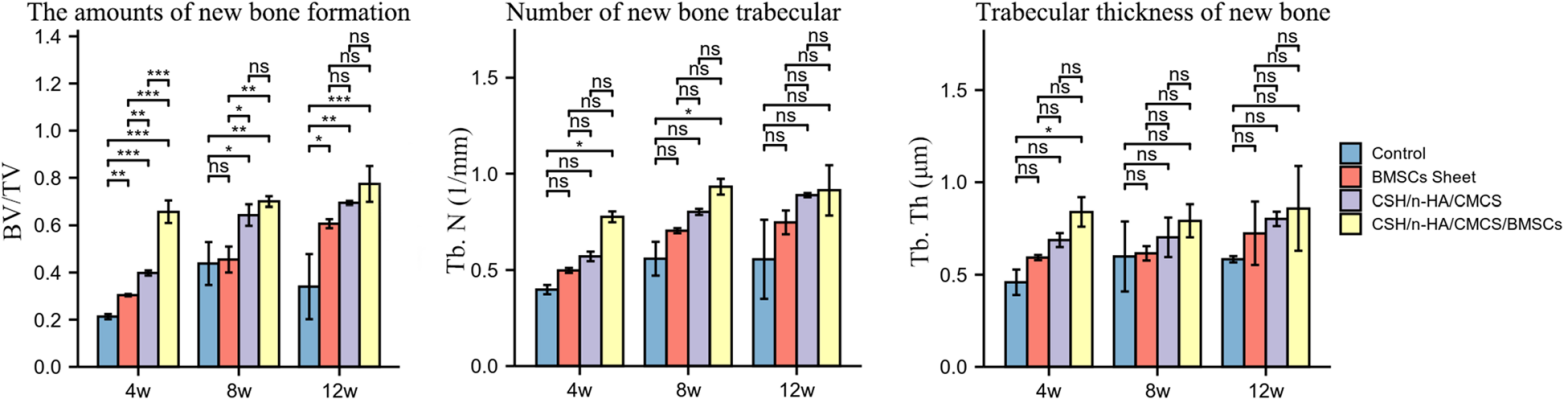
Quantitative analysis of newborn bone tissue was performed in the four groups at 4 weeks, 8 weeks and 12 weeks after the operation (ns, *p* ≥ 0.05; *, *p* < 0.05; **, *p* < 0.01; ***, *p* < 0.001). At 4 weeks, there was a statistically significant difference in new bone formation among the four groups, while up to 12 weeks, there was no difference among the three Groups B, C, and D

Micro-CT scans were obtained at 4 weeks, 8 weeks, and 12 weeks postoperatively. Overall, in Group A, the defects still existed with almost no new bone formation, while in Group B, calluses formed with densities similar to that of the surrounding bone tissue, and the defect cavities were partly treated (Fig. 9a, b). In Groups C and D, the hydrogels were gradually absorbed over time, and new bone formation was almost complete at 12 weeks (Fig. 9c, d). Moreover, by the 3D reconstruction of the target positions, material degradation and new bone formation in each group were assessed and evaluated quantitatively (Fig. 10). Four weeks after surgery, distinct cavities could be noticed in all groups, and callus formation was observed in Groups B, C, and D. In general, the BV/TV, Tb. N, Tb. Th of the four groups showed significant differences with *P* < 0.05 (*P* = 0.016, *P* = 0.016, *P* = 0.016), while only the BV/TV was statistically significant between groups. Statistical results showed that at 4 weeks, the BV/TV of the experimental group was better than those of the other three groups and especially better than that of Group C. At 8 weeks, although the defects of the control group and the BMSC sheets group tended to shrink, they showed typical radiographic symptoms of chronic osteomyelitis. As the material degraded, most of the defects were repaired and filled with novel bone in the VA/CSH/n-HA/CMCS and VA/CSH/n-HA/CMCS/BMSC sheets groups, with no recurrence of infection. Statistical analysis showed that in general, there were differences in the BV/TV and Tb. N of the four groups (*P* = 0.030, *P* = 0.016), and the BV/TV of Group D was significantly different from those of Groups A and B, while it was not different from that of Group C. At 12 weeks postoperatively, chronic osteomyelitis still existed in the control and BMSC sheets groups with cavities, rim osteosclerosis, and ossification. All materials degraded, and novel bones filled the defect completely in the VA/CSH/n-HA/CMCS and VA/CSH/n-HA/CMCS/BMSC sheets groups, as shown in Fig. 10. In general, the BV/TV values of the four groups showed a significant difference with *P* = 0.016, while the Tb. N and Tb. Th of the four groups showed no differences (*P* = 0.055, *P* = 0.218). Furthermore, there were no significant differences in new bone formation among Groups B, C, and D.

#### Van Gieson staining

**Fig. 11:**
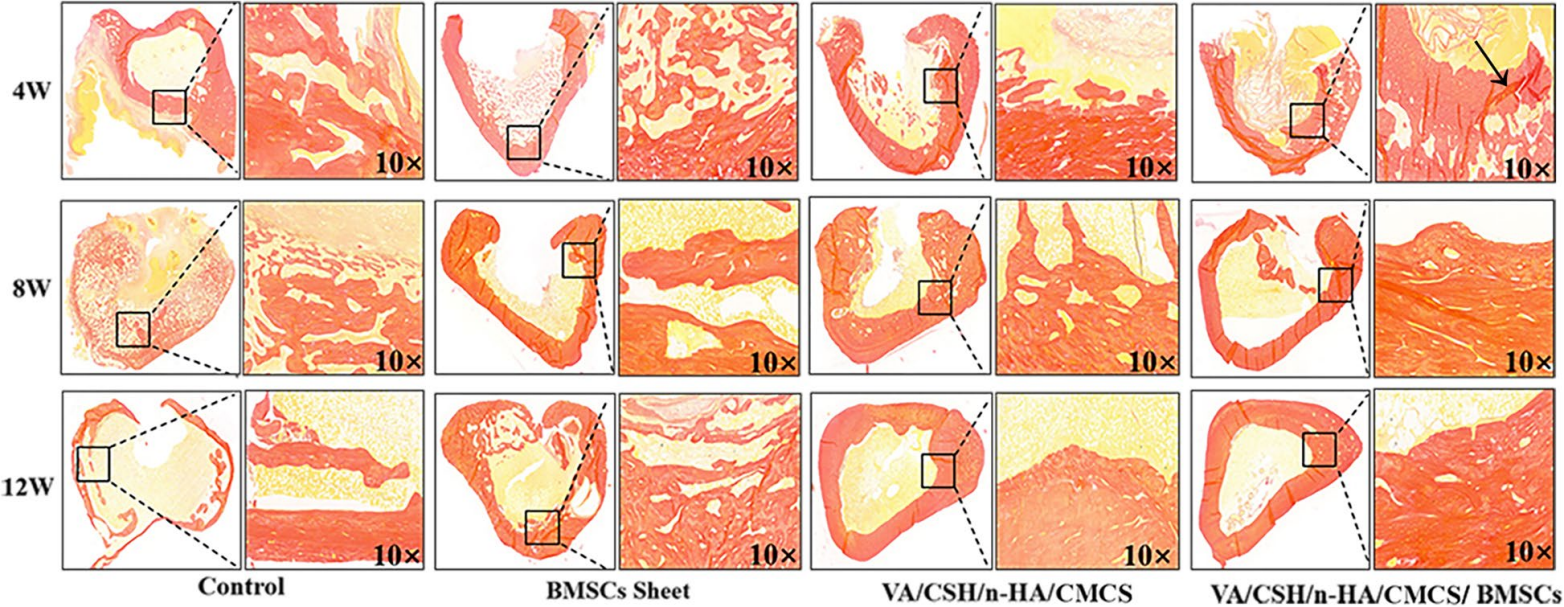
Histological observation of the four groups at 4, 8, and 12 weeks after implantation (Van Gieson staining, 10×)

Four weeks postoperatively, the VG staining images showed clear boundaries of the bone formation areas in the four groups (Fig. 11). No new collagen fibres formed in Group A, and a small amount of collagen fibres formed in Groups B, C, and D. Hydrogels were partially absorbed in Groups C and D, with a sharp interface between bone tissue. At 8 weeks, compared with Groups A and B, Groups C and D showed a number of collagen fibres, accompanied by decreased defects and novel bone formation. At 12 weeks postoperatively, defects were still visible in Group A, which contained only small amounts of collagen fibres. Defects were partially healed in Group B, with disordered bone formation. Defects were completely reconstructed in Groups C and D, of which we could see rich collagen fibres in Group D.

**Fig. 12:**
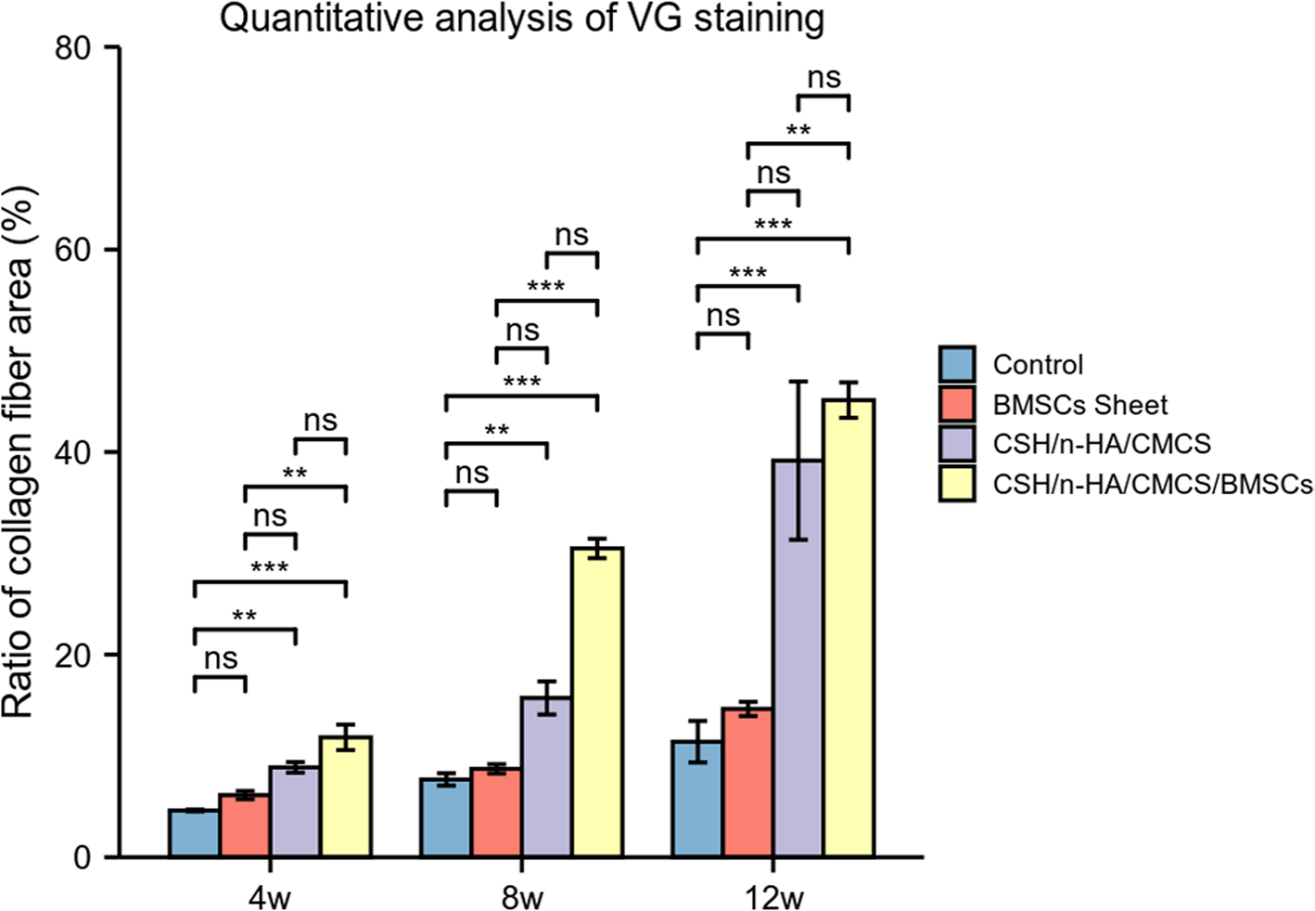
Quantitative analysis of VG staining of each group at 4, 8, and 12 weeks after treatment (ns, *p* ≥ 0.05; *, *p* < 0.05; **, *p* < 0.01; ***, *p* < 0.001)

By a quantitative analysis of the target position, the ratio of collagen fibres area (%) in each group was assessed and evaluated (Fig. 12). The results of the Kruskal-Wallis Test showed that at 4, 8, and 12 weeks, the differences of variables among the 4 groups were statistically significant (*P* < 0.001). 4 weeks after surgery, the ratio of collagen fibres area between Groups A and C, Groups A and D, Groups B and D showed significant differences with *P*<0.01 (*P* = 0.001, *P* = 0.000, *P* = 0.002). At the time point of 8 weeks, the ratio of collagen fibres area between Groups A and C, Groups A and D, Groups B and D showed significant differences with *P*<0.01 (*P* = 0.003, *P* = 0.000, *P* = 0.001). At 12 weeks postoperatively, the ratio of collagen fibres area between Groups A and C, Groups A and D, Groups B and D showed significant differences with *P*<0.05 (*P* = 0.000, *P* = 0.000, *P* = 0.010).

#### Haematoxylin and eosin staining

**Fig. 13:**
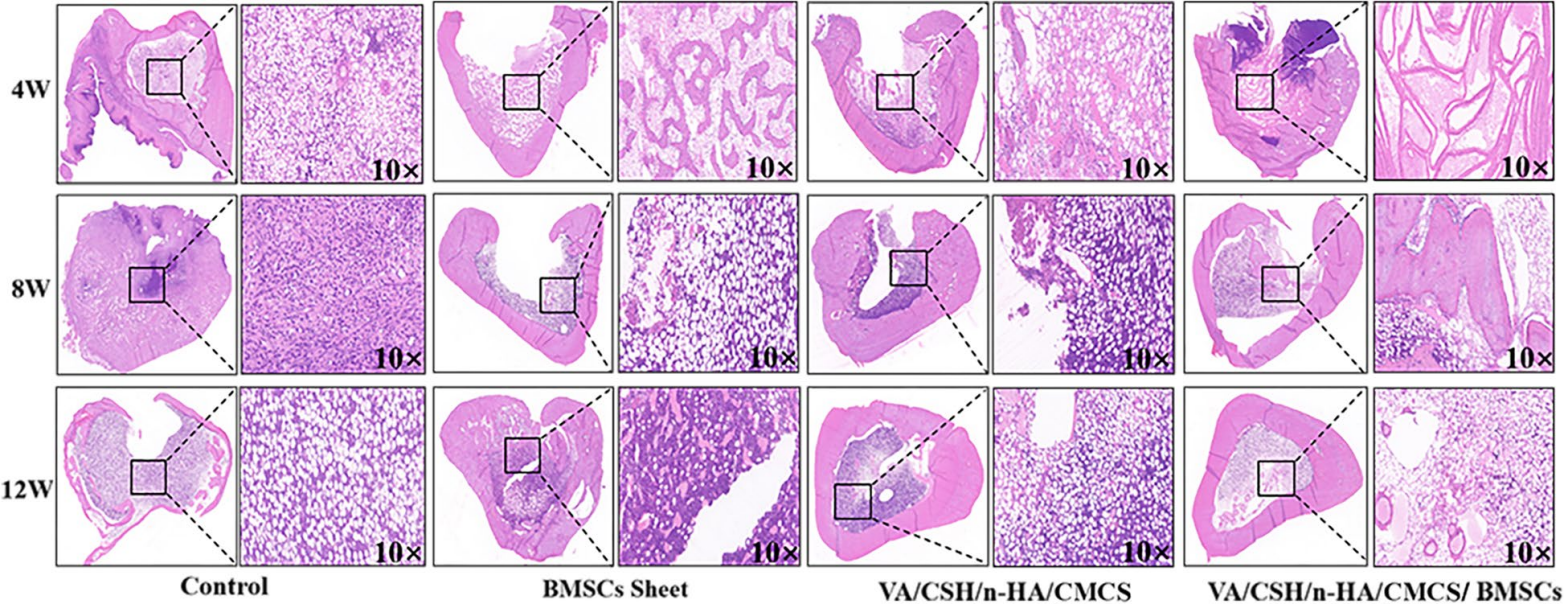
Haematoxylin-eosin (HE) staining results of the target sites of the right tibia of each group at 4, 8, and 12 weeks after treatment (10×)

HE staining was performed to identify the characteristics of the experimental sites of the four groups to further evaluate the contributions of different factors to infection control. Plentiful inflammatory cells were observed in both the control group and Group B, indicating that infection was not effectively eliminated. In the VA/CSH/n-HA/CMCS and VA/CSH/n-HA/CMCS/BMSC sheets groups, inflammatory cells decreased gradually over time, suggesting that the antibiotic loading system was effective in treating infection (Fig. 13).

**Fig. 14:**
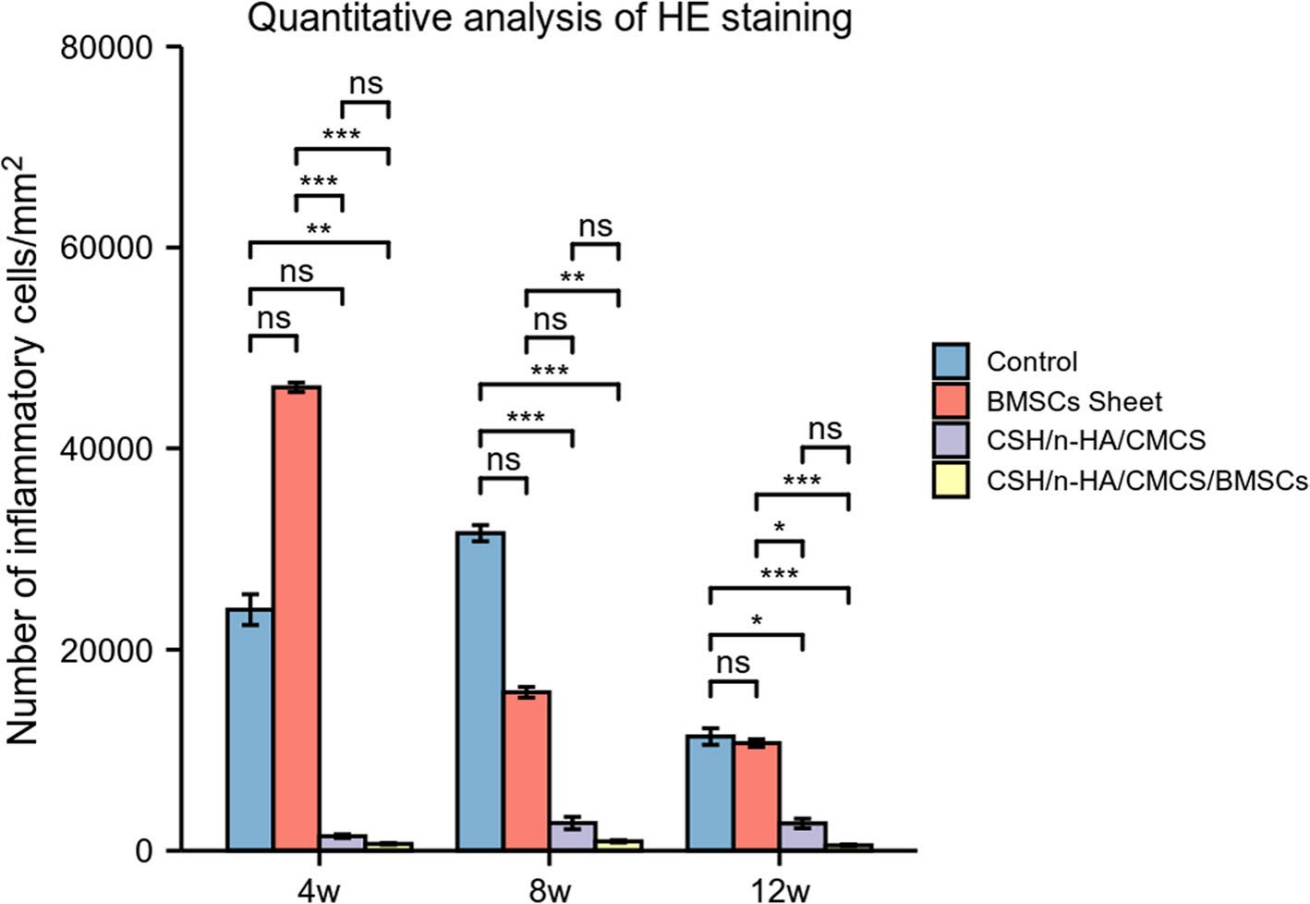
Quantitative analysis of HE staining of each group at 4, 8, and 12 weeks after treatment (ns, *p* ≥ 0.05; *, *p* < 0.05; **, *p* < 0.01; ***, *p* < 0.001)

By a quantitative analysis of the target position, the number of inflammatory cells/mm^2^ in each group was assessed and evaluated (Fig. 14). The results of the Kruskal-Wallis Test showed that at 4, 8, and 12 weeks, the differences of variables among the 4 groups were statistically significant (*P* < 0.001). 4 weeks after surgery, the number of inflammatory cells between Groups A and D, Groups B and C, Groups B and D showed significant differences with *P*<0.01 (*P* = 0.004, *P* = 0.001, *P* = 0.000). At the time point of 8 weeks, the number of inflammatory cells between Groups A and C, Groups A and D, Groups B and D showed significant differences with *P*<0.01 (*P* = 0.001, *P* = 0.000, *P* = 0.005). At 12 weeks postoperatively, the number of inflammatory cells between Groups A and C, Groups A and D, Groups B and C, Groups B and D showed significant differences with P<0.05 (*P* = 0.030, *P* = 0.000, *P* = 0.045, *P* = 0.000).

## Discussion

Thorough debridement is fundamental to effective therapy for chronic osteomyelitis [26, 27]. However, bone defects lacking vascularization make the curative effects of systemic antibiotics unsatisfactory, with the high recurrence of infections. To address these defects, autologous or allogeneic bone is traditionally chosen, while problems such as limited source, complications of the donor site, and disease transmission remain insurmountable [28]. Thus, the demand for synthetic scaffold materials for bone tissue engineering has been increasing for years, as they offer a more promising strategy for overcoming the conundrums of IBD treatment that plague orthopaedic surgeons [2]. It has been reported that a combination of appropriate inorganic materials and BMSCs may have the potential for bone regeneration [29]. The ideal injectable bone tissue engineering material should exhibit the following characteristics: excellent biocompatibility and biodegradability; capacities for bone induction and bone conduction; a proper in vivo degradation rate that matches the pace of osteogenesis; and an appropriate structure and porosity for the ingrowth of blood vessels and cell distribution [30, 31].

BMSC sheet technology overcomes the problem of the low osteogenic ability of BMSC suspensions by maintaining ECM and cell-to-cell communication, and since its development, sheet technology has been successfully applied in soft tissue reconstruction, bone defect repair, and other fields [32, 33]. The rationality for choosing CSH/n-HA/CMCS as a scaffold for BMSC sheets in our research could be explained as follows: this material has good biomechanical properties and compatibility, which has been confirmed by previous studies. Moreover, Cabañas MV et al. and Frame JW have shown in their studies that a mix of CSH and n-HA demonstrated improved injection performance compared to its respective materials [34–37]. In addition, CMCS exhibits inhibitory effects on microorganisms and activation and chemotaxis effects on macrophages [38, 39]. When combined with synthetic bone substitutes, CMCS provides space for the growth of new bone and vessels, and with a change in surrounding temperature, CMCS can be transformed from a liquid phase to a solid phase [40].

Therefore, the combination of CSH/n-HA/CMCS hydrogels, which are able to release vancomycin and promote osteogenesis, with BMSC sheets rich in ECM as well as various growth factors tends to be a good choice for the treatment of IBDs caused by chronic osteomyelitis, which may be an ideal method for repairing defects and controlling infections effectively.

In our study, infection was observed to be effectively controlled in the CSH/n-HA/CMCS group and CSH/n-HA/CMCS/BMSC sheets group during the 12-week follow-up, while chronic osteomyelitis still existed in the control group and BMSC sheets group, which was also verified by HE staining. Upon analysing the reasons for this, we reasoned that on the premise of effective debridement, the presence of a persistent cavity was not conducive to the elimination of infection. Despite the application of the BMSC sheets in Group B, which enables bone formation, this treatment could not provide a continuous defect filling effect and physical support characteristics, leading to the presence of infection. In contrast, due to the introduction of CSH/n-HA/CMCS hydrogels in Groups C and D, the absorption and degradation rates of the material were suitable so that new defects caused by excessive absorption would not be generated. Through clinical research, studies have shown that the selection of bone substitutes with appropriate degradation rates plays a significant role in the treatment of chronic osteomyelitis [41]. Moreover, the capacity of the antibiotic loading of CSH enabled the local area to maintain a high concentration of antibiotics for a long time. Effective infection control provided a prerequisite for defect reconstruction. In addition, vancomycin was selected as a locally released antibiotic because of its effectiveness against the common pathogenic bacteria of osteomyelitis (MSSA and MRSA) and its low cytotoxicity to surrounding tissues [42]. Studies on the local drug concentration release curve of vancomycin showed that the concentration dropped slowly and lasted for a long period [43].

Through 3D micro-CT reconstruction, we found that new bone sufficiently formed to repair the defects in both Groups C and D, which was significantly different from the other two groups at 4 weeks. Moreover, the capacity of bone formation at 4 weeks in Group D was better than that in Group C, which was also proven by quantitative analysis; however, at 8 and 12 weeks, there was no difference in bone formation between the two groups, indicating that the application of BMSC sheets in Group D might promote the rate of osteogenesis within 4 weeks. However, further study is still needed. Interestingly, in the control group, the amount of new bone formation increased from Week 4 to Week 8 but decreased from Week 8 to Week 12. Upon analysing the reasons for this, we reasoned that at 8 weeks, due to the persistence of infection, partial inflammatory calluses formed, and at 12 weeks, a large amount of osteonecrosis led to a reduction in the new bone formation observed during the quantitative calculation of 3D reconstruction.

VG staining showed that the content of collagen fibres in Group D was higher at the same period, and it was closely crosslinked with the scaffold material. However, at 12 weeks, there was no difference in the bone mass between Groups C and D. The high porosities of the hydrogels provided plenty of space for the ingrowth of new bone and blood vessels and surface area for direct cell adhesion and proliferation [44]. In addition, observation by gross anatomy revealed that the composite material partly degraded in approximately 4 weeks and almost entirely in 8 weeks. Reasons for this could be analysed and associated with the introduction of CSH, as it was reported that the degradation rate of CSH varied from 1.5 to 3 months in vivo and that the ratio of CSH and n-HA directly affected the structures of the hydrogels as well as their degradation speeds, which will lend itself as the direction for our future research [11, 45]. The technique of locally minimally invasive BMSC acquisition, separation, and cultivation into sheets in vitro would significantly reduce the demand for autologous bone, which could cause certain collateral damage.

By analysing the underlying mechanisms of the experimental phenomena, we believe that inorganic scaffolds with the slow release of antibiotics play a significant role in the reconstruction of IBDs, and the three-dimensional structure formed by the scaffolds provides room for cells to obtain nutrients and metabolize, which also directly affects the growth, proliferation, and migration of cells. The composite of n-HA and CSH improved upon the poor injection performance of pure n-HA, overcame the shortcomings of the fast degradation rate of CSH, as well as the slow degradation rate of n-HA, and exhibited brand-new physical and chemical properties and more optimized biological properties. In addition, the introduction of n-HA prolonged the period of antibiotic release compared to CSH alone, and the addition of CMCS with good biocompatibility also played a role in prolonging the antibiotic release time. Moreover, we found that the effect of the BMSC sheets as a single component in the treatment of IBDs was not satisfactory, which was also confirmed by the outcome of Group B. Because the osteogenesis in Group D was more obvious than that in Group C at 4 weeks, the high proliferation and multidirectional differentiation potential provided by the BMSC sheets and the ECM rich in growth factors were also particularly important; nevertheless, the deeper mechanism of its interaction with the scaffold material still needs to be explored.

However, our study has some shortcomings. Our study was designed for the reconstruction of focal IBDs, without considering the treatment of segmental IBDs, and lacked real-time monitoring and evaluation of the degradation of synthetic hydrogels in vivo, which would be beneficial for the optimization of the ratio of the different materials in hydrogels to improve their performance. Moreover, the biomechanical analysis of the hydrogels, local drug elution kinetics monitoring and pharmacokinetic characteristics after loading with vancomycin still need to be explored. In addition, hydrogels combined with BMSCs should also be used as a control group, and further validation in large animal models (sheep or pigs) is a prerequisite for preclinical study in the future.

## Conclusion

This technique of combining organic (CMCS and BMSC sheets) and inorganic materials (CSH/n-HA) not only overcame the drawbacks of the respective materials but also resulted in a material that demonstrated proper osteogenesis ability. Moreover, it showed excellent biocompatibility and biodegradability in the process of bone defect reconstruction of IBDs. The introduction of CSH equipped the hydrogels with characteristics of antibiotic release, which played an irreplaceable role in the elimination of infection, and the addition of BMSC sheets may accelerate the rate of bone formation. VA-loaded hydrogels, as bone substitutes and drug delivery carriers, provide a new approach for IBD therapy.

## Supplementary Information

**Additional file 1.**

**Additional file 2.**

**Additional file 3.**

## Data Availability

Readers can access the data and material supporting the conclusions of the study by contacting Yanjun Wang at 648943498@qq.com.

## Abbreviations

IBDs: Infected bone defects
CSH: Calcium sulfate hemihydrate
CS: Calcium sulfate
n-HA: nano-hydroxyapatite
CMCS: Carboxymethyl chitosan
BMSC: Bone marrow mesenchymal stem cell
HE: hematoxylin and eosin
VG: Van Gieson
ECM: Extracellular matrix
ALP: Alkaline phosphatase
SEM: Scanning electron microscopy

## References

[1] Aufricht G (1940). Dental molding compound cast and adhesive strapping in rhinoplastic surgical procedure. Arch Otolaryngol.

[2] Pearson JJ, Gerken N, Bae C, Lee KB, Satsangi A, McBride S (2020). In vivo hydroxyapatite scaffold performance in infected bone defects. J Biomed Mater Res.

[3] Malkawi H, Shannak A, Sunna’ P (1984). Active treatment of segmental defects of long bones with established infection. A prospective study. Clin Orthop Relat Res.

[4] Haas DW, McAndrew MP (1996). Bacterial osteomyelitis in adults: evolving considerations in diagnosis and treatment. Am J Med.

[5] Corona PS, Altayó M, Amat C, Vicente M, Velez R (2021). Reconstruction of infected post-traumatic bone defects of the distal femur with the compress implant. Preliminary results of a staged non-biological strategy. Injury.

[6] Xiao H, Wang S, Wang F, Dong S, Shen J, Xie Z. Locking compression plate as an external fixator for the treatment of tibia infected bone defects. Z Orthop Unfall. 2021. 10.1055/a-1545-5363. 10.1055/a-1545-5363 34496424

[7] Zhao ZH, Wang GL, Zhang Y, Luo W, Liu SY, Zeng ZH (2020). Induced membrane technique combined with antibiotic-loaded calcium sulfate–calcium phosphate composite as bone graft expander for the treatment of large infected bone defects: preliminary results of 12 cases. Ann Transl Med.

[8] Kimna C, Deger S, Tamburaci S, Tihminlioglu F (2019). Chitosan/montmorillonite composite nanospheres for sustained antibiotic delivery at post-implantation bone infection treatment. Biomed Mater.

[9] Betz RR (2002). Limitations of autograft and allograft: new synthetic solutions. Orthopedics.

[10] Jiang Y, Qin HJ, Wan HY, Yang J, Yu Q, Jiang M (2020). Asprin-loaded strontium-containing α-calcium sulphate hemihydrate/nano-hydroxyapatite composite promotes regeneration of critical bone defects. J Cell Mol Med.

[11] Kelly CM, Wilkins RM, Gitelis S, Hartjen C, Watson JT, Kim PT (2001). The use of a surgical grade calcium sulfate as a bone graft substitute: results of a multicenter trial. Clin Orthop Relat Res.

[12] Boukari Y, Qutachi O, Scurr DJ, Morris AP, Doughty SW, Billa N (2017). A dual-application poly (dl-lactic-co-glycolic) acid (PLGA)-chitosan composite scaffold for potential use in bone tissue engineering. J Biomater Sci Polym Ed.

[13] Shamekhi MA, Rabiee A, Mirzadeh H, Mahdavi H, Mohebbi-Kalhori D, Eslaminejad MB (2017). Fabrication and characterization of hydrothermal cross-linked chitosan porous scaffolds for cartilage tissue engineering applications. Mater Sci Eng C Mater Biol Appl.

[14] Okano T, Yamada N, Sakai H, Sakurai Y (1993). A novel recovery system for cultured cells using plasma-treated polystyrene dishes grafted with poly (N-isopropylacrylamide). J Biomed Mater Res.

[15] Lu Y, Zhang W, Wang J, Yang GZ, Yin S, Tang TT (2019). Recent advances in cell sheet technology for bone and cartilage regeneration: from preparation to application. Int J Oral Sci.

[16] Ito M, Shichinohe H, Houkin K, Kuroda S (2017). Application of cell sheet technology to bone marrow stromal cell transplantation for rat brain infarct. J Tissue Eng Regen Med.

[17] Xu M, Li J, Liu X, Long SQ, Shen Y, Li Q (2019). Fabrication of vascularized and scaffold-free bone tissue using endothelial and osteogenic cells differentiated from bone marrow derived mesenchymal stem cells. Tissue Cell.

[18] Kobayashi J, Akiyama Y, Yamato M, Shimizu T, Okano T (2018). Design of temperature-responsive cell culture surfaces for cell sheet-based regenerative therapy and 3D tissue fabrication. Adv Exp Med Biol.

[19] Manialopoulos C, Scdek J, Meleher AH (1988). Bone formation in vitro by stromal cells obtained from bone marrow of young adult rats. Cell Tissue Res.

[20] Deng LZ, Liu Y, Yang LQ, Yi JZ, Deng FL, Zhang LM (2020). Injectable and bioactive methylcellulose hydrogel carrying bone mesenchymal stem cells as a filler for critical-size defects with enhanced bone regeneration. Colloids Surf B Biointerfaces.

[21] Naudot M, Garcia Garcia A, Jankovsky N, Barre A, Zabijak L, Zakaria Azdad S (2020). The combination of a poly-caprolactone/nano-hydroxyapatite honeycomb scaffold and mesenchymal stem cells promotes bone regeneration in rat calvarial defects. J Tissue Eng Regen Med.

[22] Walsh WR, Chapman-Sheath PJ, Cain S, Debes J, Bruce WJM, Svehla MJ (2003). A resorbable porous ceramic composite bone graft substitute in a rabbit metaphyseal defect model. J Orthop Res.

[23] Fang B, Qiu P, Xia C, Cai D, Zhao CC, Chen Y (2021). Extracellular matrix scaffold crosslinked with vancomycin for multifunctional antibacterial bone infection therapy. Biomaterials.

[24] Broussou DC, Lacroix MZ, Toutain PL, Woehrlé F, Garch FE, Bousquet-Melou A (2018). Differential activity of the combination of vancomycin and amikacin on planktonic vs biofilm-growing Staphylococcus aureus bacteria in a hollow fiber infection model. Front Microbiol.

[25] Norden CW, Kennedy E, Experimental osteomyelitis. I. (1970). A description of the model. J Infect Dis.

[26] Drampalos E, Mohammad HR, Pillai A (2020). Augmented debridement for implant related chronic osteomyelitis with an absorbable, gentamycin loaded calcium sulfate/hydroxyapatite biocomposite. J Orthop.

[27] Bibbo C, Stough JD (2012). Reduction calcaneoplasty and local muscle rotation flap as a salvage option for calcaneal osteomyelitis with soft tissue defect. J Foot Ankle Surg.

[28] Ueha T, Akahane M, Shimizu T, Uchihara Y, Morita Y, Nitta N (2015). Utility of tricalcium phosphate and osteogenic matrix cell sheet constructs for bone defect reconstruction. World J Stem Cells.

[29] Liang J, Li W, Zhuang N, Wen SN, Huang SJ, Lu WZ (2021). Experimental study on bone defect repair by BMSCs combined with a light-sensitive material: g-C_3_N_4_/rGO. J Biomater Sci Polym Ed..

[30] Gupta P, Adhikary M, M JC, Kumar M, Bhardwaj N, Mandal BB (2016). Biomimetic, osteoconductive non-mulberry silk fiber reinforced tricomposite scaffolds for bone tissue engineering. ACS Appl Mater Interfaces.

[31] Camarero-Espinosa S, Rothen-Rutishauser B, Weder C, Foster EJ (2016). Directed cell growth in multi-zonal scaffolds for cartilage tissue engineering. Biomaterials.

[32] Tang Z, Okano T (2014). Recent development of temperature-responsive surfaces and their application for cell sheet engineering. Regen Biomater.

[33] Ma D, Ren L, Chen F, Liu Y, Zhang J, Xue Z (2010). Reconstruction of rabbit critical-size calvarial defects using autologous bone marrow stromal cell sheets. Ann Plast Surg.

[34] Nilsson M, Wang JS, Wielanek L, Tanner KE, Lidgren L (2004). Biodegradation and biocompatability of a calcium sulphate-hydroxyapatite bone substitute. J Bone Joint Surg Br.

[35] Delloye C, Cnockaert N, Cornu O (2003). Bone substitutes in 2003: an overview. Acta Orthop Belg.

[36] Frame JW, Rout PGJ, Browne RM (1987). Ridge augmentation using solid and porous hydroxylapatite particles with and without autogenous bone or plaster. J Oral Maxillofac Surg.

[37] Cabanas MV, Rodriguez-Lorenzo LM, Vallet-Regi M (2002). Setting behavior and in vitro bioactivity of hydroxyapatite/calcium sulfate cements. Chem Mater.

[38] Patel VR, Amiji MM (1996). Preparation and characterization of freeze-dried chitosan-poly (ethylene oxide) hydrogels for site-specific antibiotic delivery in the stomach. Pharm Res.

[39] Noel SP, Courtney H, Bumgardner JD, Haggard WO (2008). Chitosan films: a potential local drug delivery system for antibiotics. Clin Orthop Relat Res.

[40] Yan Y, Zhang X, Li C, Huang Y, Ding Q, Pang X (2015). Preparation and characterization of chitosan-silver/hydroxyapatite composite coatings onTiO_2_ nanotube for biomedical applications. Appl Surf Sci.

[41] Zhao ZH, Wang GL, Zhang Y, Luo W, Liu SY, Liu YY (2020). The effect of calcium sulfate/calcium phosphate composite for the treatment of chronic osteomyelitis compared with calcium sulfate. Ann Palliat Med.

[42] Colding-Rasmussen T, Horstmann P, Petersen MM, Hettwer W (2018). Antibiotic elution characteristics and pharmacokinetics of gentamicin and vancomycin from a mineral antibiotic carrier: an in vivo evaluation of 32 clinical cases. J Bone Jt Infect.

[43] Nugent M, McLaren A, Vernon B, McLemore R (2010). Strength of antimicrobial bone cement decreases with increased poragen fraction. Clin Orthop Relat Res.

[44] Ma PX (2004). Scaffolds for tissue fabrication. Mater Today.

[45] Roberts TT, Rosenbaum AJ (2012). Bone grafts, bone substitutes and orthobiologics: the bridge between basic science and clinical advancements in fracture healing. Organogenesis.

